# Zonal Soil Type Determines Soil Microbial Responses to Maize Cropping and Fertilization

**DOI:** 10.1128/mSystems.00075-16

**Published:** 2016-07-12

**Authors:** Mengxin Zhao, Bo Sun, Linwei Wu, Qun Gao, Feng Wang, Chongqing Wen, Mengmeng Wang, Yuting Liang, Lauren Hale, Jizhong Zhou, Yunfeng Yang

**Affiliations:** a State Key Joint Laboratory of Environment Simulation and Pollution Control, School of Environment, Tsinghua University, Beijing, China; b State Key Laboratory of Soil and Sustainable Agriculture, Institute of Soil Science, Chinese Academy of Sciences, Nanjing, China; c Ningbo Academy of Agricultural Sciences, Ningbo, China; d Department of Microbiology and Plant Biology and Institute for Environmental Genomics, University of Oklahoma, Norman, Oklahoma, USA; e Fisheries College, Guangdong Ocean University, Zhanjiang, China; f Earth Sciences Division, Lawrence Berkeley National Laboratory, Berkeley, California, USA; Chinese Academy of Sciences

**Keywords:** zonal soil type, microbial community, fertilization, soil functional process, GeoChip

## Abstract

Soil types heavily influence ecological dynamics. It remains controversial to what extent soil types shape microbial responses to land management changes, largely due to lack of in-depth comparison across various soil types. Here, we collected samples from three major zonal soil types spanning from cold temperate to subtropical climate zones. We examined bacterial and fungal community structures, as well as microbial functional genes. Different soil types had distinct microbial biomass levels and community compositions. Five years of maize cropping (growing corn or maize) changed the bacterial community composition of the Ultisol soil type and the fungal composition of the Mollisol soil type but had little effect on the microbial composition of the Inceptisol soil type. Meanwhile, 5 years of fertilization resulted in soil acidification. Microbial compositions of the Mollisol and Ultisol, but not the Inceptisol, were changed and correlated (*P* < 0.05) with soil pH. These results demonstrated the critical role of soil type in determining microbial responses to land management changes. We also found that soil nitrification potentials correlated with the total abundance of nitrifiers and that soil heterotrophic respiration correlated with the total abundance of carbon degradation genes, suggesting that changes in microbial community structure had altered ecosystem processes.

**IMPORTANCE** Microbial communities are essential drivers of soil functional processes such as nitrification and heterotrophic respiration. Although there is initial evidence revealing the importance of soil type in shaping microbial communities, there has been no in-depth, comprehensive survey to robustly establish it as a major determinant of microbial community composition, functional gene structure, or ecosystem functioning. We examined bacterial and fungal community structures using Illumina sequencing, microbial functional genes using GeoChip, microbial biomass using phospholipid fatty acid analysis, as well as functional processes of soil nitrification potential and CO_2_ efflux. We demonstrated the critical role of soil type in determining microbial responses to land use changes at the continental level. Our findings underscore the inherent difficulty in generalizing ecosystem responses across landscapes and suggest that assessments of community feedback must take soil types into consideration.

**Author Video**: An author video summary of this article is available.

## Abstract (precis)

Microbial communities are essential drivers of soil functional processes such as nitrification and heterotrophic respiration. Although there is initial evidence revealing the importance of soil type in shaping microbial communities, there has been no in-depth, comprehensive survey to robustly establish it as a major determinant of microbial community composition, functional gene structure, or ecosystem functioning. We examined bacterial and fungal community structures using Illumina sequencing, microbial functional genes using GeoChip, microbial biomass using phospholipid fatty acid analysis, as well as functional processes of soil nitrification potential and CO_2_ efflux. We demonstrated the critical role of soil type in determining microbial responses to land use changes at the continental level. Our findings underscore the inherent difficulty in generalizing ecosystem responses across landscapes and suggest that assessments of community feedback must take soil types into consideration.

## INTRODUCTION

Soils are heterogeneous, with distinctive characteristics in relation to various parent materials and influences from past and present climatic conditions (1). Soil taxonomy classifies soils at six levels, i.e., orders, suborders, great groups, subgroups, families, and series (2, 3). The formation of zonal soils is classified primarily by climate, whereas intrazonal and azonal soils are classified by local factors such as parent material (4, 5). Black (Mollisol), Chao (Inceptisol), and red (Ultisol) soils are three main zonal soil types in East and Southeast Asia. Of these three types, Mollisols are mainly distributed in the northeastern region of China belonging to cold temperate zones, which is characterized by high fertility and agricultural productivity (6). Inceptisols are distributed in warm temperate zones, such as South Korea and the Yellow River basin of China (7, 8). Ultisols are the most widespread soils of Southeast Asia in the middle subtropical zone, accounting for 51% of the region (8). 

A number of studies have analyzed microbial community compositions in Mollisols, Inceptisols, and Ultisols (9 – 11). However, most of these studies used low-resolution techniques, such as denaturing gradient gel electrophoresis (DGGE) and clone library analyses (10, 12), or focused on a few microbial functional groups, such as methanogens, nitrifiers, denitrifiers, and straw decomposers (9, 11). For several studies to examine the effect of soil type on bacterial diversity, ammonia-oxidizing bacteria and archaea, rhizosphere bacteria, and fungi (13 – 16), a prevailing observation was that soil type was a principal driver in shaping microbial community composition.

Plant crops substantially affects soil microbial communities, owing to direct and indirect influences, such as organic matter inputs and root exudates (17). Seven years of maize cropping (growing maize or corn) increased organic carbon and total nitrogen content in the Inceptisol soil type, as well as microbial biomass of *Actinobacteria*, *Bacteriodetes*, *Acidobacteria*, and *Alphaproteobacteria* (18). A laboratory experiment with maize litter amendment to three different soils (Agrudalf, Hapludalf, and Xerochrept) revealed consistent increases in bacterial diversity (19). However, another laboratory incubation experiment showed that plant cropping caused disparate effects on microbial community diversity and composition across soil types (sand, sandy loam, and clay) (20). To date, it remains unclear whether microbes respond similarly to plant cropping across soil types under *in situ* field conditions.

The effect of nitrogen, phosphate, and potassium (NPK) fertilization on microbial communities has been well documented (21 – 24). A recent nitrogen and phosphorus addition experiment under laboratory conditions showed consistent microbial responses to nutrient input across global grassland soils (25). In contrast, *in situ* observations were more variable, as no significant change in microbial community composition was detected in a 55-year NPK fertilization experiment (23), but microbial community composition was altered by 16-year, 22-year, and 150-year NPK fertilization treatments (21, 22, 26). Similarly, a 20-year experiment revealed significant effects of manure and nitrogen fertilizers on bacterial community abundance and composition (24). It is still unclear whether the inconsistent findings are caused by differences in soil types, fertilization regimes, cropped plants, abundance of life history strategists, or analytical techniques used to assess the microbial communities.

To address the aforementioned uncertainty, here we report a parallel, holistic survey of microbial communities in Mollisol, Inceptisol, and Ultisol soils using integrated, high-throughput molecular technologies. We aim to address the following questions. (i) Does maize cropping impose consistent effects on microbial communities and soil processes across zonal soil types? (ii) Does NPK fertilization impose consistent effects on microbial communities and soil processes across zonal soil types? Our results demonstrated that microbial community compositions substantially differed in all three zonal soil types studied. Also, maize cropping and fertilization were inconsistent in their effects on microbial communities, which could be attributed to variations in microbial life history strategies and/or environmental selection.

## RESULTS

### Environmental variables.

Zonal soil types were distinct in environmental variables and functional processes (see Table S1 in the supplemental material). Notably, soil organic matter (SOM) in the Mollisol soil type was more than 4 times higher than those in the Inceptisol and Ultisol soil types. The total nitrogen (TN) and the nitrate (NO_3_-N) contents in the Mollisol were twice as large as those in the Inceptisol and Ultisol. The ammonium (NH_4_-N) content in the Ultisol was 1.8 mg/kg, about twofold higher than those in the Mollisol and Inceptisol. Nitrification potential and CO_2_ efflux in the Ultisol were substantially lower than those in the Mollisol and Inceptisol (Table S1).

10.1128/mSystems.00075-16.8 Table S1  Summary of environmental and microbial variables. Download Table S1, DOCX file, 0.02 MB. Copyright © 2016 Zhao et al. 2016 Zhao et al. This content is distributed under the terms of the Creative Commons Attribution 4.0 International license.

Most environmental variables were changed by maize cropping, but soil pH, NH_4_-N, and NO_3_-N remained unchanged in any soil type (see Table S1 in the supplemental material). Nitrogen, phosphorus, and potassium (NPK) fertilization significantly decreased cation exchange capacity (CEC) in all three soil types. It also decreased soil pH by 0.4 in the Mollisol and Inceptisol and by 0.1 in the Ultisol. In addition, SOM, available phosphorus (AP), NH_4_-N, and NO_3_-N were significantly increased in the Mollisol.

### Soil microbial communities.

Total, bacterial, and fungal biomass of the soil microbial communities in the Mollisol were at least twice as large as those in the other two soil types (see Table S1 in the supplemental material). Maize cropping increased bacterial and fungal biomass in the Inceptisol but did not affect bacterial or fungal biomass in the Mollisol or Ultisol. NPK fertilization decreased bacterial biomass by 20.4% and fungal biomass by 42.6% in the Mollisol and increased total biomass by 72.3% in the Ultisol.

Both detrended correspondence analysis (DCA) and hierarchical clustering analysis showed that microbial communities were clustered based on soil types (see Fig. S1 in the supplemental material). However, maize cropping changed only bacterial community composition in the Ultisol and fungal community composition and functional gene structure in the Mollisol. NPK fertilization changed bacterial community composition in the Mollisol and Ultisol and fungal community composition and functional composition in the Mollisol.

10.1128/mSystems.00075-16.1 Figure S1  Microbial community compositions in three soil types. The percentage of variation explainable by each axis is shown. (A) Detrended correspondence analysis (DCA) of the bacterial community; (B) hierarchical clustering analysis of the bacterial community; (C) DCA of the fungal community; (D) hierarchical clustering analysis of the fungal community; (E) DCA of functional genes; (F) hierarchical clustering analysis of functional genes. Biological replicates clustered in the hierarchical clustering analysis are framed to indicate changes in microbial community composition by maize cropping or NPK fertilization. Download Figure S1, TIF file, 2.8 MB. Copyright © 2016 Zhao et al. 2016 Zhao et al. This content is distributed under the terms of the Creative Commons Attribution 4.0 International license.

The multiple regression tree (MRT) analyses showed that bacterial, fungal, and functional compositions were primarily influenced by soil types (see Fig. S2 in the supplemental material), suggesting that maize cropping and NPK fertilization had relatively minor influences on microbial communities. Further analyses showed that the contributions of soil types to the total variations in microbial communities were higher (67.6% for bacteria and 16.8% for fungi) than those of maize cropping (6.8% for bacteria and 15.3% for fungi) and NPK fertilization (5.9% for bacteria and 11.2% for fungi).

10.1128/mSystems.00075-16.2 Figure S2  Multiple regression tree analyses to determine the relative importance of the soil type, maize crop, and NPK fertilization in affecting bacterial community composition at the OTU level (A), fungal community composition at the OTU level (B), and functional community composition at the functional gene level (C). Each split minimizes the variation of the data within a node and maximizes the variation between nodes. The three study sites had Mollisol, Inceptisol, and Ultisol soil types. Download Figure S2, TIF file, 0.7 MB. Copyright © 2016 Zhao et al. 2016 Zhao et al. This content is distributed under the terms of the Creative Commons Attribution 4.0 International license.

**FIG 1:**
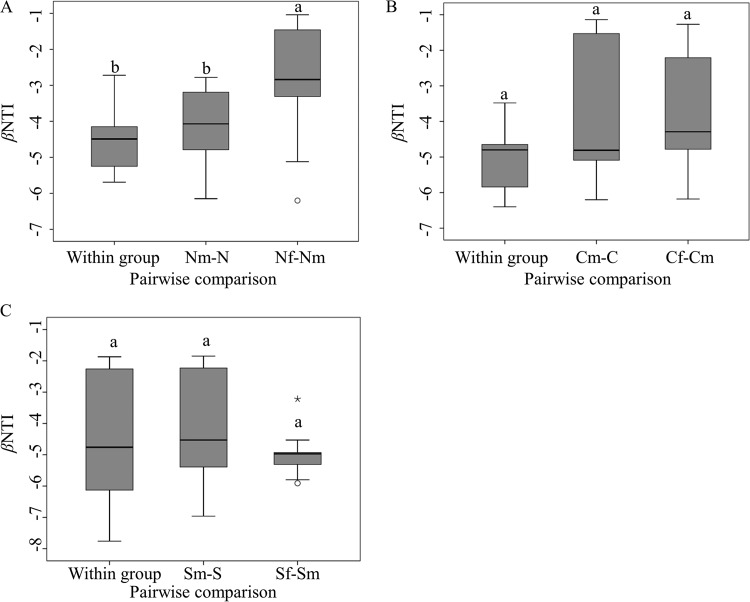
Boxplots of βNTI distribution in the Mollisol (A), Inceptisol (B), and Ultisol (C) soil types. Each boxplot shows the median value (thick black line), first quartile (bottom of the box), third quartile (top of the box), and range of the data that were no more than 1.5 times height of the boxes (error bars). Moderate outliers (circles) and extreme outlier (asterisk) are also shown. Significance was determined by one-way ANOVA followed by the LSD test. Boxes with the same letter were not statistically significantly different (*P* > 0.05). Boxes of within groups represent pairwise comparisons between any two samples within a treatment. Boxes for Nm-N, Cm-C, and Sm-S represent pairwise comparisons between maize cropping samples (m suffix) and bare fallow samples (no suffix) for samples from sites N, C, and S. Boxes of Nf-Nm, Cf-Cm, and Sf-Sm represent pairwise comparisons between NPK fertilization samples (f suffix) and maize cropping samples (m suffix) from the three sites.

Phylogenetic information from sequencing data can be used to calculate the β-nearest taxon index (βNTI) (see Materials and Methods for details). A |βNTI| of <2 indicates an insignificant deviation between observed and expected phylogenetic turnover and hence the dominance of stochastic processes (27), while a |βNTI| of >2 indicates the dominance of deterministic processes (28). We found that βNTIs were smaller than −2 in all three soil types, suggesting the dominance of deterministic processes in shaping microbial communities (Fig. 1). Maize cropping did not change βNTI in any soil type. However, NPK fertilization significantly increased βNTIs in the Mollisol but not the other two soils, suggesting that the effect of NPK fertilization on phylogenetic turnover was disparate in those three soil types.

### Specific taxon groups.

Different microbial taxa were found in the soil types. At the phylum level, *Verrucomicrobia* amounted to 22.8% of taxa in the Mollisol, which was 5.6- and 1.9-fold greater than their abundance in the Inceptisol and Ultisol (see Fig. S3A in the supplemental material). At the genus level, none of the top 10 abundant genera in each soil type was shared by all three soil types (Fig. S3B and S3C). For example, *Spartobacteria* was the most abundant bacterial genus in the Mollisol (19.9%) and Ultisol (10.8%) but accounted for only 1.1% of total abundance in the Inceptisol. Subgroup 6 (Gp6) and Gp4 of *Acidobacteria* accounted for more than 5.0% of the total abundance in the Mollisol and Inceptisol, while they were only 0.6% and 0.3% in the Ultisol, respectively.

10.1128/mSystems.00075-16.3 Figure S3  Distribution of bacterial phylum abundance (A), 10 most abundant bacterial genera (B), and 10 most abundant fungal genera (C). Heat map was generated with gplots package in R 2.15.0. The numbers in the heat maps show the ranks of genus abundance in each soil type. Download Figure S3, TIF file, 2.1 MB. Copyright © 2016 Zhao et al. 2016 Zhao et al. This content is distributed under the terms of the Creative Commons Attribution 4.0 International license.

Maize cropping had little effect on bacterial or fungal phyla in the Mollisol and Inceptisol (see Fig. S4A and S4B in the supplemental material), while *Alphaproteobacteria*, *Betaproteobacteria*, *Gammaproteobacteria*, *Gemmatimonadetes*, and *Nitrospira* were increased in the Ultisol (Fig. S4A). At the genus level, no bacterial or fungal genera in all three soil types showed consistent responses to maize cropping except *Acidobacteria* Gp7, which increased by more than 18.0% in all three soil types (Table S2).

10.1128/mSystems.00075-16.4 Figure S4  Fold changes of microbial relative abundance in soil with a maize crop or NPK fertilization. (A) Bacterial changes in soil with a maize crop; (B) fungal changes in soil with a maize crop; (C) bacterial changes in fertilized soil; (D) fungal changes in fertilized soil; (E) fold changes of carbon decomposing enzymes in soil with a maize crop; (F) fold changes of carbon decomposing enzymes in fertilized soil. Results are shown as average values of three biological replicates with error bars of standard deviations. Significance was determined by unpaired *t* test in Microsoft Excel 2013. Values that are significantly different are indicated by asterisks as follows: *, *P* < 0.05; **, *P* < 0.01; ***, *P* < 0.001. Download Figure S4, TIF file, 2.4 MB. Copyright © 2016 Zhao et al. 2016 Zhao et al. This content is distributed under the terms of the Creative Commons Attribution 4.0 International license.

10.1128/mSystems.00075-16.9 Table S2  Relative abundance of genus that consistently responded to maize cropping (Gp7) or fertilization (Gp4, Gp6, and *Fusarium*). Download Table S2, DOCX file, 0.02 MB. Copyright © 2016 Zhao et al. 2016 Zhao et al. This content is distributed under the terms of the Creative Commons Attribution 4.0 International license.

**FIG 2:**
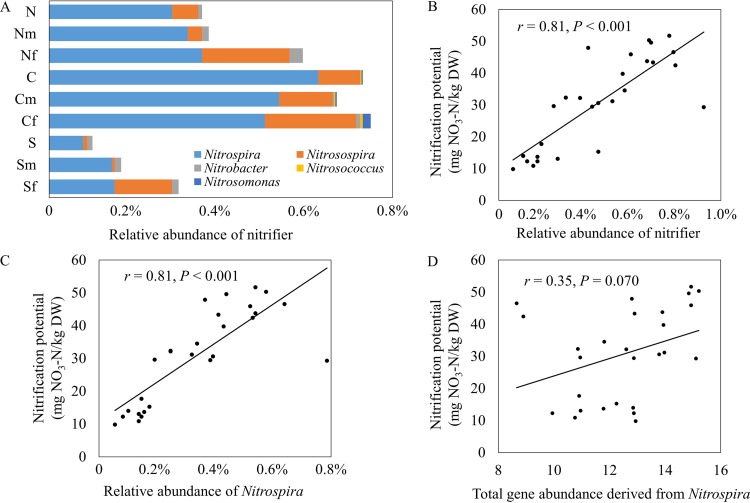
Abundances of nitrifiers and their relationships with nitrification potentials. (A) Distribution of nitrifier abundances. The N, C, and S abbreviations refer to sample sites, and the suffix of “m” and “f” indicate maize cropping and NPK fertilization, respectively. (B) Pearson correlation between the total abundance of nitrifiers and nitrification potential. (C) Pearson correlation between the relative abundance of *Nitrospira* and nitrification potential. (D) Pearson correlation between gene abundance derived from *Nitrospira* in GeoChip and nitrification potential. The nitrification potential shown in panels B to D is shown per kilogram of weight (dry weight [DW]). Correlation *r* and *P* were determined by Pearson correlation and TDIST tests, respectively.

No consistent effect of NPK fertilization on bacterial or fungal community composition was observed in all three soil types (see Fig. S4C and S4D in the supplemental material). At the phylum level, the only exception was *Acidobacteria*, which marginally (*P* = 0.070) decreased (Fig. S4C). At the genus level, only two bacterial genera (*Acidobacteria* Gp4 and *Acidobacteria* Gp6) decreased and one fungal genus (*Fusarium*) increased across all three soil types (Table S2). Five detected genera were related to nitrification (nitrifier), in which *Nitrospira* was the most abundant one and *Nitrosospira* was marginally (*P* = 0.100) increased by NPK fertilization in all three soil types (Fig. 2A).

### Carbon and nitrogen cycling genes.

A total of 26,493 distinct genes associated with carbon and nitrogen cycling were detected by GeoChip analyses. The levels of nitrogenase-encoding genes (*nifH*) and ammonia monooxygenase-encoding genes (*amoA*) were lower in the Inceptisol soil type than in the Mollisol and Ultisol soil types (see Fig. S5A in the supplemental material). In contrast, the levels of genes involved in the denitrification processes, such as the *narG* gene encoding a nitrate reduction enzyme and the *nosZ* gene encoding a nitrous oxide reduction enzyme, were higher in the Inceptisol.

10.1128/mSystems.00075-16.5 Figure S5  Relative nitrogen cycling gene abundances in nitrogen cycling (A) and their responses to maize crop and NPK fertilization (B). Total gene abundances were divided by their probe numbers to generate relative abundance. Results are shown as average values of three biological replicates with standard deviations indicated by the error bars. Treatments with the same letters are not significantly different (*P* > 0.05), as determined by one-way ANOVA, followed b they LSD test in SAS version 6.1. Download Figure S5, TIF file, 1.7 MB. Copyright © 2016 Zhao et al. 2016 Zhao et al. This content is distributed under the terms of the Creative Commons Attribution 4.0 International license.

Maize cropping and NPK fertilization caused distinct changes in the levels of carbon and nitrogen cycling genes among all three soil types (see Fig. S5B in the supplemental material). The levels of almost all genes involved in denitrification were decreased by maize cropping in the Inceptisol (Fig. S5B). In contrast, the levels of *NifH* and *amoA* genes were increased in the Mollisol and Inceptisol and decreased in the Ultisol. The levels of the *norB* (encoding nitric oxide reductase) and *hao* (encoding hydroxylamine oxidoreductase) genes were also increased by NPK fertilization in the Ultisol, but not in the other two soil types.

### Linkages between microbial community composition and environmental variables.

To examine environmental factors in shaping bacterial and fungal community composition, we performed Mantel tests with microbial communities and environmental variables in three bare fallow soils (see Table S3 in the supplemental material). Bacterial community composition was significantly correlated with soil pH, soil bulk density (BD), soil porosity (Sp), total phosphorus (TP), total potassium (TK), NH_4_-N, annual average temperature, annual rainfall, and relative humidity. Similarly, a significant (*P* = 0.035) model of canonical correspondence analysis (CCA) also showed that NH_4_-N, BD, TK and annual rainfall correlated with bacterial community composition (Fig. S6A). Fungal community composition correlated with soil pH, TK, NH_4_-N, and annual rainfall, as revealed by Mantel tests (Table S3) and CCA (Fig. S6B).

10.1128/mSystems.00075-16.6 Figure S6  Canonical correspondence analysis (CCA) to determine the main variables shaping the bacterial community (A) and the fungal community (B). Environmental variables were selected by variance inflation factors (VIF) of less than 20. Abbreviations: N, C, and S, N, C, and S sample sites, respectively; Ec, electrical conductivity; BD, soil bulk density; TK, total potassium; AK, available potassium; NH_4_-N, ammonium nitrogen; NO_3_-N, nitrate; Annual R, annual rainfall. Download Figure S6, TIF file, 0.3 MB. Copyright © 2016 Zhao et al. 2016 Zhao et al. This content is distributed under the terms of the Creative Commons Attribution 4.0 International license.

10.1128/mSystems.00075-16.10 Table S3  Significance of correlations between environmental variables and microbial communities in bare fallow soils determined by Mantel tests. Download Table S3, DOCX file, 0.02 MB. Copyright © 2016 Zhao et al. 2016 Zhao et al. This content is distributed under the terms of the Creative Commons Attribution 4.0 International license.

Mantel tests were also carried out to identify major environmental variables that explain changes in microbial community composition by maize cropping and NPK fertilization. Soil pH, CEC, TP, and the seed weight of maize were correlated with bacterial communities (*r* > 0.34; *P* < 0.052) in the Mollisol, while only seed weight correlated with bacterial communities (*r* = 0.31; *P* = 0.049) in the Inceptisol (Table 1). Eight variables (soil pH, CEC, TP, electrical conductivity [EC], AP, NO_3_-N, seed weight, and aboveground biomass of maize) correlated (*r* > 0.50; *P* < 0.012) with bacterial communities in the Ultisol.

**TABLE 1:** Correlations between environmental variables and microbial and fungal communities^a^

Environmental variable^b^	Correlation^c^ **between the environmental variable and the following community:**											
	Bacterial						Fungal					
	N site		C site		S site		N site		C site		S site	
	*r*	*P*	*r*	*P*	*r*	*P*	*r*	*P*	*r*	*P*	*r*	*P*
pH	0.34	**0.052**	−0.16	0.680	0.40	**0.024**	−0.01	0.481	−0.16	0.662	0.17	0.232
SOM	0.17	0.155	0.17	0.246	0.36	0.058	0.27	0.083	0.60	0.078	0.07	0.352
WHC	−0.18	0.882	0.13	0.333	0.20	0.125	0.23	0.094	0.74	0.102	−0.05	0.617
BD	−0.14	0.835	0.00	0.489	0.15	0.159	0.32	**0.049**	0.56	0.106	−0.06	0.627
Sp	−0.14	0.807	−0.06	0.598	0.15	0.188	0.32	**0.031**	0.42	0.130	−0.12	0.679
EC	0.03	0.439	0.24	0.136	0.76	**0.001**	0.05	0.382	0.14	0.234	0.20	0.161
CEC	0.67	**0.005**	−0.03	0.460	0.75	**0.001**	−0.07	0.651	−0.03	0.435	0.19	0.194
TN	−0.21	0.854	0.11	0.334	−0.36	0.978	0.29	0.058	0.34	0.177	−0.19	0.65
TP	0.36	**0.039**	−0.21	0.749	0.44	**0.037**	−0.04	0.525	−0.13	0.591	−0.15	0.657
TK	−0.11	0.719	−0.09	0.614	0.24	0.142	−0.15	0.783	−0.01	0.316	0.10	0.336
AP	−0.03	0.521	−0.16	0.776	0.50	**0.012**	0.18	0.179	−0.12	0.628	−0.10	0.613
AK	0.23	0.136	0.29	0.068	−0.26	0.937	0.27	0.076	0.10	0.214	−0.20	0.76
NH_4_-N	0.14	0.203	−0.19	0.728	0.09	0.324	−0.13	0.708	−0.18	0.671	0.23	0.156
NO_3_-N	−0.01	0.500	0.22	0.122	0.72	**0.001**	−0.26	0.874	0.43	**0.037**	0.15	0.248
Seed wt	0.49	**0.010**	0.31	**0.049**	0.70	**0.003**	0.02	0.368	0.24	0.078	0.08	0.33
Aboveground biomass	0.20	0.126	0.20	0.092	0.69	**0.002**	0.17	0.174	0.15	0.162	0.08	0.331

a The correlations between environmental variables and the bacterial or fungal community were determined by the Mantel test. Each site has bare fallow samples, maize cropping samples, and NPK fertilization samples. The N, C, and S sites have Mollisol, Inceptisol, and Ultisol soil types, respectively.

b Abbreviations: SOM, soil organic matter; WHC, water holding capacity; BD, soil bulk density; Sp, soil porosity; EC, electrical conductivity; CEC, cation exchange capacity; TN, total nitrogen; TP, total phosphorus; TK, total potassium; AP, available phosphorus; AK, available potassium; seed wt, seed weight.

c Correlations that are significant (*P* < 0.050) are indicated by boldface type.

Only BD (*r* = 0.32; *P* = 0.049) and Sp (*r* = 0.32;*P* = 0.031) correlated with fungal communities in the Mollisol, while only NO_3_-N (*r* = 0.43; *P* = 0.037) correlated with fungal communities in the Inceptisol. No linkages between environmental variables and fungal communities were observed in the Ultisol. We found a positive correlation between BD and the abundance of strictly anaerobic bacteria *Clostridium* (*r* = 0.56; *P* = 0.002) (see Fig. S7A in the supplemental material). In addition, βNTI correlated with CEC (*r* = −0.43; *P* = 0.009) and AP (*r* = 0.48; *P* = 0.003) in the Mollisol (Fig. S7B and S7C) and with NH_4_-N (*r* = −0.47; *P* = 0.004) in the Ultisol (Fig. S7D).

10.1128/mSystems.00075-16.7 Figure S7  Pearson correlation between environmental variables and microbial communities. (A) Soil bulk density (BD) and total abundance of *Clostridium*; (B) cation exchange capacity in the Mollisol and βNTI; (C) available phosphorus in the Mollisol and βNTI; (D) NH_4_-N in the Ultisol and βNTI. Correlation *r* and *P* were determined by Pearson correlation and TDIST tests, respectively. Download Figure S7, TIF file, 0.8 MB. Copyright © 2016 Zhao et al. 2016 Zhao et al. This content is distributed under the terms of the Creative Commons Attribution 4.0 International license.

### Linkages between microbial communities and soil functional processes.

We found that the total abundance of nitrifiers and the subgroup of *Nitrospira* (29) strongly and positively (*r* = 0.81; *P* < 0.001) correlated with nitrification potentials (Fig. 2B and C). Furthermore, there was a positive correlation (*r* = 0.35; *P* = 0.070) between genes derived from *Nitrospira* and nitrification potentials (Fig. 2D), suggesting that sequencing and GeoChip data were largely consistent.

**FIG 3:**
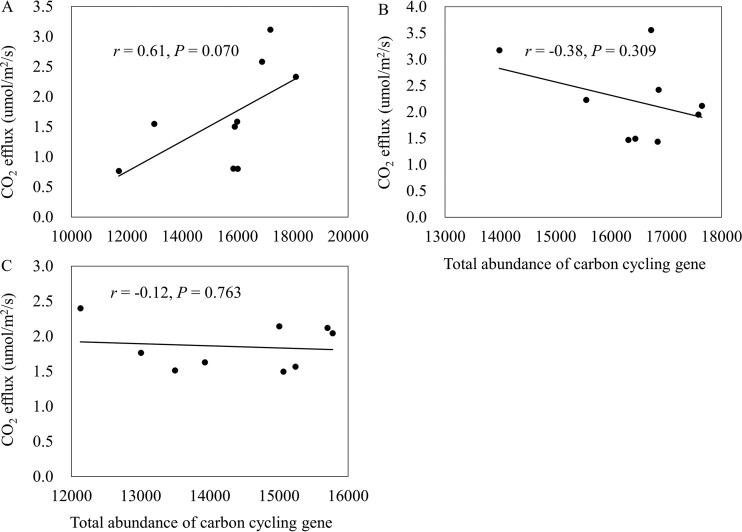
Pearson correlations between CO_2_ efflux and total carbon cycling gene abundance in bare fallow soil (A), soil where maize was grown (B), and (C) NPK-fertilized soil. Correlation *r* and *P* were determined by Pearson correlation and TDIST tests, respectively.

Soil heterotrophic respiration is an ecological consequence of microbial activities. Accordingly, we detected a modest, positive correlation (*r* = 0.61; *P* = 0.070) between the abundance of carbon cycling genes and CO_2_ efflux in bare fallow soils (Fig. 3A), but not in soils with a maize crop or fertilized soils (*r* > −0.38; *P* > 0.309) (Fig. 3B and C), which was consistent with our recent study (30).

## DISCUSSION

In this study, we carried out Illumina MiSeq sequencing and GeoChip experiments to analyze microbial community compositions and functional potentials in three major soil types and their responses to maize cropping and NPK fertilization. We found that microbial communities substantially differed in soil types (see Fig. S1 in the supplemental material). Furthermore, microbial community responses to maize cropping or NPK fertilization varied by soil type (Fig. S4).

The importance of soil type as the main factor in shaping microbial community composition has been well documented (13, 16), with findings that *Verrucomicrobia* was the most abundant bacterial phylum and *Fusarium* was the most abundant fungal genus in a Mollisol of northeastern China and that *Penicillium* and *Aspergillus* were among the most prevalent fungal genera in an Ultisol (12, 31). Our results provided supporting evidence for these findings (26). In addition, we found that the *Curvularia* fungus, which is commonly found in warm environments (32), was also abundant in the Ultisol. The substantial differences in climate and soil environments of all soil types might give rise to very different microbial community compositions with no overlap among the top 10 abundant genera for each soil type (see Fig. S1 and Fig. S3 in the supplemental material).

Five years of maize cropping and NPK fertilization altered microbial community compositions (see Fig. S1 in the supplemental material). Interestingly, we observed few similar changes in DNA abundance among all three soil types. There were several potential alternative explanations.

Bacteria fall into two main life history strategies, copiotrophs or oligotrophs (33), similar to the *r*-selected or *K*-selected strategies in plants and animals. In general, *r*-strategists grow faster and turn over more rapidly, while *K*-strategists propagate in longer time intervals and acclimate more rapidly (34). Nutrient stimuli could affect populations in ways that shift dominance to organisms with advantageous life history strategies. Nitrogen fertilization could cause a shift in life history strategy, which stimulates copiotrophic microbes but not oligotrophic microbes (35, 36). For example, it was noted that *Proteobacteria*, *Bacteroidetes*, and *Actinobacteria*, three copiotrophic groups, increased in abundance by long-term nitrogen addition in a grassland and in an agricultural field (36), while the fungal *Chytridiomycota*, of which most genera were oligotrophic groups (37), showed little response to nitrogen amendments (38). Exogenous nitrogen input accelerated labile carbon decomposition but inhibited recalcitrant carbon decomposition, which was mediated by copiotrophic and oligtrophic microbes, respectively (35). NPK fertilization consistently resulted in an increase in the abundance of taxon groups dominated by copiotrophic bacteria, such as *Proteobacteria* and *Actinobacteria*, in the Mollisol in our study, and a decrease in abundance of generally oligotrophic microbes, such as the bacterial taxa *Acidobacteria* and *Verrucomicrobia* (see Fig. S4C in the supplemental material) and fungal taxa of *Chytridiomycota* and *Zygomycota* (Fig. S4D). In addition, copiotrophic classes of *Beta*- and *Gammaproteobacteria* were also increased by fertilization in the Mollisol, while an oligotrophic *Verrucomicrobia* genus *Spartobacteria* was decreased in the Mollisol and Ultisol.

As fast-growing microbes are less efficient at substrate use than slow-growing ones, the average microbial biomass of fast-growing bacteria, such as *Alpha*- and *Gammaproteobacteria*, tends to be smaller (33), Therefore, a shift from oligotrophs to copiotrophs effectively reduces total microbial biomass. We consistently found that NPK fertilization decreased bacterial and fungal biomass in the Mollisol (see Table S1 in the supplemental material). In addition, our sequencing results differed from a recent study that microbial communities across global-scale grasslands revealed consistent responses to N and P fertilization (25). A close examination showed that soil samples in the study of Leff et al. (25) had a narrow pH range (mostly between 5.1 and 6.7) and only slight soil acidification by fertilization, which resulted in no correlation between soil pH and any of the major bacterial taxa. In contrast, soil pH spanned a large range from 4.9 to 8.1 in our study (Table S1). Many studies have attributed the effect of inorganic nitrogen fertilization on soil microbes to soil acidification (18, 39). For example, addition of ammonium sulfate led to soil acidification and an almost 10-fold decrease of a dominant bacterial phylum in an Inceptisol (18). In this study, NPK fertilization decreased the soil pH by 0.4 and 0.1 unit in the Mollisol and Ultisol, respectively, which was consistent with previous studies showing that nitrogen fertilization led to soil acidification (24, 40). Thus, soil pH might be the main factor causing shifts in the microbial communities in these two soil types, which was confirmed by the Mantel tests showing positive correlations between bacterial communities and soil pH (*r* > 0.34; *P* < 0.050) (Table 1). In contrast, when soil pH decreased from 8.1 to 7.7 in the Inceptisol (Table S1), no significant correlation between soil pH and bacterial community (*r* = 0.16; *P* = 0.68) was detected (Table 1), verifying that a shift to neutral pH was inconsequential for most bacteria (41, 42). In addition, fungal communities were less sensitive to soil pH than bacterial communities, owing to their wider growth tolerances to pH range (43, 44). Accordingly, no significant correlation between pH and fungal communities was detected in any soil type (Table 1).

Soil physical variables are important in shaping microbial community composition but are often underestimated by microbiologists owing to lack of physical variable measurements (45). Since low BD or high Sp increases soil aeration (46), anaerobic microbes are generally inhibited, which explained our observation of a positive correlation (*r* = 0.56; *P* = 0.002) between obligate anaerobic *Clostridium* and BD (see Fig. S7A in the supplemental material). The influence of BD and Sp on microbial community was further verified by their correlations with fungal community in the Mollisol (Table 1). In addition, a strong positive correlation (*r* = 0.76; *P* < 0.001) between bacterial composition in the Ultisol and EC, known to impose strong influence on microbial community composition (47 – 49), was observed (Table 1).

In summary, we examined microbial community compositions and their responses to maize cropping and NPK fertilization in three zonal soil types. Generally, soil type overrode maize cropping or NPK fertilization as the main determinant of microbial community compositions and soil variables. In addition, maize cropping or NPK fertilization caused disparate changes in the composition of the microbial communities or functional gene structures, which endorses the importance of taking soil type in consideration when examining ecosystem responses to global changes.

## MATERIALS AND METHODS

We conducted this study at three long-term experimental stations well maintained by the Chinese Academy of Sciences. The study station located in Hailun, Heilongjiang Province, China (E126°38′ and N47°26′) (designated the N site) has a cold temperate monsoon climate, and the soil type is Mollisol. The study station located in Fengqiu, Henan Province, China (E114°24′ and N35°00′) (designated the C site) has a warm temperate monsoon climate, and the soil type is Inceptisol. The study station located in Yingtan, Jiangxi Province, China (E116°55′ and N28°15′) (designated the S site) has a middle subtropical monsoon climate, and the soil type is Ultisol.

The sites were established in October 2005 as plots that were 1.4 m by 1.2 m by 1.0 m. Maize or corn was grown in triplicate plots every year since the spring of 2006, with subtypes of Haiyu 6 at the N site, Zhengdan 958 at the C site, and Denghai 11 at the S site. Maize cropping, together with fertilizers of CO(NH_2_)_2_, (NH_4_)_2_HPO_4_, and KCl at the level of 150 kg N, 75 kg P_2_O_5_ and 60 kg K_2_O per ha, was administered to another triplicate plots. The P and K fertilizers and half of the amount of the N fertilizers were applied before maize cropping (growing maize). The other half of the N fertilizer was applied at the maize bell stage. Bare fallow plots were used as a control for maize cropping and NPK fertilization.

We collected soil samples within 2 days after harvesting maize in August and September 2011. We took 10 soil cores of 2-cm diameter at a depth of 0 to 15 cm from each plot and combined them. Soil for geochemical analyses was kept on ice during transport and stored at 4°C in the laboratory. Soil for DNA analyses was kept in liquid nitrogen during transport and stored at −80°C in the laboratory. Samples were designated by the sites. The “m” suffix indicated maize cropping, and the “f” suffix indicated NPK fertilization, while bare fallow samples had no suffix.

### Environmental variable measurements.

Details of environmental variable measurements were described in our previous studies (30). In brief, we measured soil organic matter (SOM) content by heated dichromate oxidation and titration with ferrous ammonium sulfate. We measured CO_2_ efflux once a week in July and August to calculate average CO_2_ efflux. We measured microbial biomass by the phospholipid fatty acid (PLFA) content using a modified Bligh-Dyer protocol as outlined in our previous studies (30, 65). We determined soil nitrification potential by an incubation method in (NH_4_)_2_SO_4_ solution (50).

### Illumina MiSeq sequencing.

We extracted microbial genomic DNA using a freeze-grinding method and purified it using 0.5% low-melting-point agarose gel electrophoresis (51). We used primers 515F (F stands for forward) (5′-GTGCCAGCMGCCGCGGTAA-3′) and 806R (R stands for reverse) (5′-GGACTACHVGGGTWTCTAAT-3′) with sample-specific bar codes and Illumina adapter sequences to target the V4 hypervariable region of bacterial 16S rRNA genes and primers gITS7F (5′-GTGARTCATCGARTCTTTG-3′) and ITS4R (5′-TCCTCCGCTTATTGATATGC-3′) to target the internal transcribed spacer II (ITS2) region of fungal ribosome encoding genes (52). DNA for Illumina sequencing was amplified by two rounds of PCR. A 25-µl PCR system containing 2.5 µl of 10× PCR buffer, 0.1 µl of high-fidelity AccuPrime *Taq* DNA polymerase (Invitrogen, Carlsbad, CA), 1 µl of each primer (10 µM), and 5 or 15 µl of template DNA was used in three technical replicates. To target the 16S rRNA genes, the first round of PCR was carried out using primers without the bar codes by the following cycling conditions: (i) an initial denaturation step of 1 min at 94°C; (ii) 10 cycles, with 1 cycle consisting of 20 s at 94°C, 25 s at 53°C, and 45 s at 68°C; (iii) a final extension step of 10 min at 68°C. PCR products from three replicates were combined and purified using an Agencourt AMPure XP kit (Beckman Coulter, Brea, CA) following the manufacturer’s instructions and eluted in 50-µl water. We used 15 µl of the purified PCR product as the template for the second round of PCR amplification using bar-coded primers in three technical replicates under the same cycling conditions as in the first round of PCR amplification and 20 cycles, rather than 10. We examined PCR products from the second round by electrophoresis with 1% agarose gel. Amplification products of three technical replicates were then combined and quantified by PicoGreen using a FLUOstar Optima microplate reader (BMG Labtech, Jena, Germany). We pooled PCR products from different samples together in equal concentrations, purified the pooled sample using Qiagen gel extraction kits (Qiagen Sciences, Germantown, MD) following the manufacturer’s instructions, and requantified using PicoGreen. Finally, we ran the purified library on MiSeq after mixing with PhiX (Illumina, San Diego, CA) at the Institute for Environmental Genomics of the University of Oklahoma (53). The amplification steps for the fungal ITS were similar to those for the 16S rRNA gene except for changes in the PCR conditions. The protocol consisted of initial denaturation of 3 min at 94°C, followed by 14 cycles for the first amplification round and 26 cycles for the second amplification round, with 1 cycle consisting of 30 s at 94°C, 30 s at 55°C, and 30 s at 68°C, and terminated with an extension step of 7 min at 68°C.

We processed raw data from Illumina sequencing of the 16S rRNA gene and fungal ITS on the Galaxy pipeline (http://zhoulab5.rccc.ou.edu) as previously described (54). We discarded low-quality sequences with nonassigned or mismatched bar codes, low-quality scores (<25), short sequence reads (<100 bp), or more than one undetermined nucleotide (N). Combined sequences with forward and reverse reads were trimmed to 245 to 260 bp for the 16S rRNA gene or to 250 to 350 bp for the fungal ITS. Sequences were classified into operational taxonomic units (OTUs) with 97% similarity for the 16S rRNA gene and 97.5% similarity for the fungal ITS after excluding chimeric sequences by using the UCHIME method (55). Singletons that were present only once across all samples were removed. Then we resampled the sequence numbers as 10,947 for the 16S rRNA gene and 9,917 for the fungal ITS, which were the minimum numbers of sequences across all samples. We assigned taxonomic information to sequences by the RDP classifier (56) for the 16S rRNA gene, and by a training set provided by the UNITE group for the fungal ITS (57). The confidence cutoff was set at 0.5. The relative abundance (RA) of sequences used in this study was calculated as $\%{\mathrm{RA}}_{ij}=\;\frac{Sij}{\Sigma_{J=\;1}^{N}Sij}\text{× }100$, where *Sij* was the sequence number of the *j*th OTU in the *i*th sample.

### GeoChip analysis.

GeoChip 4.6 is a microbial functional gene array that allows for the simultaneous assessment of more than 410 gene families essential to nutrient biogeochemical cycling and various other environmentally significant microbial functions. GeoChip 4.6 experiments were conducted as previously described (58, 59). In brief, purified DNA was labeled with the fluorescent nucleic acid dye Cy5 and then hybridized on GeoChip 4.6 slides. Slides were scanned with a scanner (MS 200 microarray scanner; NimbleGen), and the signal intensity of each spot was quantified with ImaGene version 6.0 (BioDiscovery, El Segundo, CA).

Raw data from GeoChip 4.6 were processed by removing spots with signal-to-noise ratio [SNR = (signal mean − background intensity)/background standard deviation] of <2.0 and singletons in triplicates. Data were then logarithmically transformed and divided by the mean value of each slide, which was referred to as gene abundance.

### Statistical analyses.

We used detrended correspondence analysis (DCA) and hierarchical clustering analysis to examine microbial community composition (60, 61). We used multiple regression tree (MRT) analysis to compare the relative importance of different treatments (62). We used Mantel tests to examine correlations of environmental variables with microbial community structures (63). We performed canonical correspondence analysis (CCA) with forward selection of environmental variables using variance inflation factors (VIF) of less than 20. We carried out these analyses with R software version 2.15.3 (R Development Core Team, R Foundation for Statistical Computing, Vienna, Austria), using vegan (v. 2.0-10) package for DCA and using hierarchical clustering analysis, Mantel tests, and mvpart (v. 1.6-1) package for MRT.

We used a null modeling approach to quantify bacterial phylogenetic turnover in each soil (64). The observed and expected abundance weighted β-mean nearest taxon distances (βNMTD) across all pairwise community comparisons within and between treatments were calculated as follows: $\mathrm{\beta NMTD}\;=\;0.5\;\;\left[\sum_{{\text{i}}_{\text{k}}=1}^{{\text{n}}_{\text{k}}}f1_{k}\min\left(\Delta i_{k}j_{m}\right)\;+\;\sum_{i_{m}=1}^{n_{m}}f1_{m}\min\left(\Delta i_{m}j_{k}\right)\right]$, where *n_k_* and *n_m_* were the number of OTUs in the communities *k* and *m*, respectively, *fi_k_* was the abundance of OTU *i* in community *k*, and min(Δ*i_k_j_m_*) was the minimal phylogenetic distance between OTU *i* in community *k* with all OTUs *j* in *m*. Then, we used the β-nearest taxon index $\left(\mathrm{\beta NTI}\right)\;\left[\text{βNTI}\;=\;\left({\mathrm{\beta NMTD}}_{\text{obs}}-\bar{{\mathrm{\beta NMTD}}_{\text{null}}}\right)/\text{SD}\left({\mathrm{\beta NMTD}}_{\text{null}}\right)\right]$, where βNMTD_obs_ and βNMTD_null_ were the observed and null values of βNMTD, respectively, and SD was the standard deviation of the null βNMTD distribution] to quantify the direction of phylogenetic turnover. The βNTI was calculated by picante (v. 1.6-2) package in R software with abundance weighted = TRUE and 999 times randomization to generate the null model.

We calculated α-diversity by Shannon diversity index (*H*) as $H=-\sum_{i=\;1}^{S}p_{i}\ln p_{i}$, where *S* was the species number and *p_i_* was the frequency of species *i*. Statistical significance of differences was determined by one-way analysis of variance (ANOVA) followed by the least significant difference (LSD) test in SAS with 95% confidence (version 6.1) (SAS Inc., Cary, NC) or two-tailed, unpaired Student’s *t* tests in Microsoft Excel. We conducted Pearson correlation or TDIST tests to determine the *r* or *P* value of linear correlations between environmental variables and microbial communities in Microsoft Excel. We used *P* < 0.05 to infer significant difference unless stated otherwise in the main text.

### Accession numbers.

Both MiSeq sequencing and GeoChip 4.0 data are available online (http://www.ncbi.nlm.nih.gov/). GeoChip 4.0 data were deposited under accession number GSE77546, and MiSeq sequencing data were deposited under accession number SRP069263.
