# Supplementary material for: Zonal Soil Type Determines Soil Microbial Responses to Maize Cropping and Fertilization
Source: mSystems. 2016 Jul 12;1(4):e00075-16. doi: 10.1128/mSystems.00075-16 (PMC5069962; doi:10.1128/mSystems.00075-16)
Supplement: Table S1 [file sys004162038st8.docx]

**Table S1** Summary of environmental and microbial variables

|  | N | Nm | Nf | C | Cm | Cf | S | Sm | Sf |
| --- | --- | --- | --- | --- | --- | --- | --- | --- | --- |
| *Soil physical-chemical variable* | | | | | | | | | |
| pH | 6.2±0.2c *^b^* | 6.1±0c | 5.7±0.2d | 7.9±0.1ab | 8.1±0.1a | 7.7±0.3b | 5.3±0.1e | 5±0.1ef | 4.9±0.1f |
| SOM*^a^* (g/kg) | 46.4±0.8c | 51.4±1.2b | 54.5±1.3a | 8.7±0.4ef | 8.3±0.5f | 9.7±1.3def | 9.8±0.1de | 9.6±0.4def | 10.7±0.3d |
| WHC (%) | 48.9±0b | 71.2±6.4a | 68.9±4.8a | 25.3±0d | 26.5±1.2d | 27.3±5.2d | 27.9±0d | 38.8±4.5c | 41.7±2.5c |
| BD (g/cm^3^) | 1.1±0c | 0.9±0d | 0.9±0.1d | 1.5±0a | 1.4±0ab | 1.4±0.1ab | 1.4±0b | 1.1±0.1c | 1.1±0c |
| Sp (%) | 56.4±0b | 65.4±0.9a | 64.4±2.4a | 45±0cd | 47.6±1.6c | 46.5±3.5cd | 43.1±0d | 52.6±3.5b | 54.1±3.8b |
| Ec (μs/cm) | 22.7±3.1e | 52.0±5.9b | 74.0±21a | 33.1±6.6de | 66.6±8.8a | 51.5±6bc | 26.7±1.8de | 37.6±1.1cd | 80.3±2.8a |
| CEC (cmol/kg) | 34.3±1.3a | 34.5±0.7a | 31.0±1b | 11.8±0.5c | 34.4±0.5a | 11.8±0.6c | 12.3±0.4c | 12.0±0.4c | 7.9±0.3d |
| TN (g/kg) | 1.8±0.3b | 2.2±0.1a | 2.3±0.2a | 0.6±0c | 0.5±0.1c | 0.8±0.2c | 0.6±0.1c | 0.8±0.4c | 0.7±0.1c |
| TP (g/kg) | 0.8±0ab | 0.8±0ab | 0.9±0a | 0.7±0.1bc | 0.6±0de | 0.7±0cd | 0.4±0f | 0.4±0f | 0.5±0ef |
| TK (g/kg) | 18.7±0.1ab | 18.4±0.5ab | 18.5±0.2ab | 20.6±3.8a | 17.5±0.4b | 17.6±0.5b | 9.7±0.5c | 9.7±0.4c | 9.3±0.4c |
| AP (mg/kg) | 39.6±3.8a | 26.8±5.6b | 37.2±4.1a | 7.3±0.3d | 3.5±0.4d | 7.8±1.2d | 17.3±0.7c | 17.3±2.6c | 29.7±3.9b |
| AK (mg/kg) | 201.7±40.2a | 139.2±5.8b | 112.5±6.6c | 99.2±7.6cd | 73.3±1.4e | 84.2±5.8de | 109.2±1.4c | 110±5c | 110.8±3.8c |
| NH_4_-N (mg/kg) | 0.6±0.4b | 0.6±0.1b | 1.6±0.8a | 0.6±0.3b | 0.8±0.1b | 0.9±0.2b | 1.8±0.5a | 1.6±0.1a | 1.7±0.3a |
| NO_3_-N (mg/kg) | 13.3±5.9ab | 10.6±6.2b | 23.3±18.5a | 4.4±1.6b | 4.2±0.5b | 6.8±1.6b | 5.3±0.3b | 5.2±0.4b | 6.6±0.3b |
| *Plant variable* | | | | | | | | | |
| seed weight (kg/ha) |  | 2246±1464d | 8652±2046a |  | 5340±225bc | 7274±854ab |  | NA*^c^* | 3955±672cd |
| above ground biomass  (kg/ha) | | 4822±284cd | 9523±3035a |  | 5232±964bc | 7477±1067ab |  | 2440±103d | 6349±343bc |
| TN_Seed (g/kg) |  | 10.7±1.7bc | 13.2±1.2a |  | 9.4±1.3c | 11.7±0.7ab |  | NA | 9.1±0.5c |
| TP_Seed (g/kg) |  | 3.7±0.8a | 4.4±0.4a |  | 1.8±0.7b | 1.9±0.4b |  | NA | 2±0.2b |
| TK_Seed (g/kg) |  | 4.5±0.6a | 5.4±0.6a |  | 3.3±0.2b | 3.5±0.4b |  | NA | 3.4±0.2b |
| TOC_Seed (g/kg) |  | 418.1±3.9ab | 417.8±12.8ab |  | 440.9±5.9a | 433.8±24.5a |  | NA | 391.3±9.2b |
| TN_Stem (g/kg) |  | 4.7±1.3b | 6.8±1.4a |  | 6.2±1a | 6.8±0.7a |  | 3.2±0.8c | 3.7±0.5bc |
| TP_Stem (g/kg) |  | 2.8±0.5a | 2±0.8b |  | 0.6±0.2c | 0.8±0.2c |  | 1.8±0.2b | 0.4±0.1c |
| TK_Stem (g/kg) |  | 5.2±3.1b | 11.6±3.3a |  | 10.6±1.9a | 14.3±2.3a |  | 13.2±0.6a | 11.6±4.9a |
| TOC_Stem (g/kg) |  | 372.9±2c | 388.3±7.9bc |  | 421.6±7a | 420.7±8.2a |  | 382.7±5.7c | 403.8±15.2b |
| *Climate parameter* | | | | | | | | | |
| Annual T (℃) | 2.1c | 2.1c | 2.1c | 13.5b | 13.5±0b | 13.5b | 18.1a | 18.1a | 18.1a |
| Annual R (mm) | 496.2c | 496.2c | 496.2c | 832.9b | 832.9b | 832.9b | 1495a | 1495a | 1495a |
| Relative humidity (%) | 64.7b | 64.7b | 64.7b | 72.0a | 72.0a | 72.0a | 61.6c | 61.6c | 61.6c |
| *Microbial biomass (nmol/g DW)* | | | | | | | | | |
| Bacterial | 12±1a | 11.4±1.7a | 9.1±0.9b | 2.1±0.5e | 4.1±1.7cd | 3.1±0.2de | 3.4±0.4de | 3.8±0.5cde | 5.4±0.5c |
| Fungal | 3.3±1ab | 3.5±1.2a | 2±0.3c | 0.7±0.1d | 2.2±0.9bc | 1.6±0.2cd | 1.8±0.5cd | 1.5±0.2cd | 2.5±0.6abc |
| Total | 42.2±8.6a | 43.1±12.7a | 35.4±4.1ab | 9.6±0.6e | 18.1±4.4cde | 16.4±1.3de | 21.3±4.8cd | 16.6±0.3de | 28.6±7.1bc |
| Fungal/bacterial | 0.3±0.1cd | 0.3±0.1cd | 0.2±0d | 0.4±0.1bcd | 0.5±0.1ab | 0.5±0.1ab | 0.5±0.2a | 0.4±0.1abc | 0.5±0.1abc |
| *Soil functional process* | |  |  |  |  |  |  |  |  |
| Nitrification (mg NO_3_-N/kg DW) | 37.5±9bc | 29.9±0.6c | 36.4±6.3bc | 41.9±11.1ab | 43.4±3.4ab | 47.9±4.9a | 12±2.1d | 12.3±1.4d | 15.4±2.3d |
| CO_2_ efflux (umol/m^2^/s) | 1063.5a | 1050.2±5.7a | 1061.1±4.5a | 512.4±85.8c | 722±92.3b | 435.5±50.5cd | 322.8±144.1d | 500.9±101.4c | 527.5±21.1c |

*^a^*Abbreviation: SOM – soil organic matter, WHC – water hold capacity, BD – soil bulk density, Sp – soil porosity, Ec – electrical conductivity, CEC – cation exchange capacity, TN – total nitrogen, TP – total phosphorus, TK – total potassium, AP – available phosphorus, AK - available potassium, TOC – total organic matter, Annual T – annual average temperature, Annual R – annual rainfall.

Values were MEAN ± SD. SD stands for standard deviation

*^b^*Letters behind each value indicate significance of differences. Treatments with any same letters are insignificantly different (*P* > 0.05) as determined by one-way ANOVA followed by the LSD test in SAS version 6.1.

*^c^*NA: data not available.
