# Supplementary figures and images for: Zonal Soil Type Determines Soil Microbial Responses to Maize Cropping and Fertilization
Source: mSystems. 2016 Jul 12;1(4):e00075-16. doi: 10.1128/mSystems.00075-16 (PMC5069962; doi:10.1128/mSystems.00075-16)

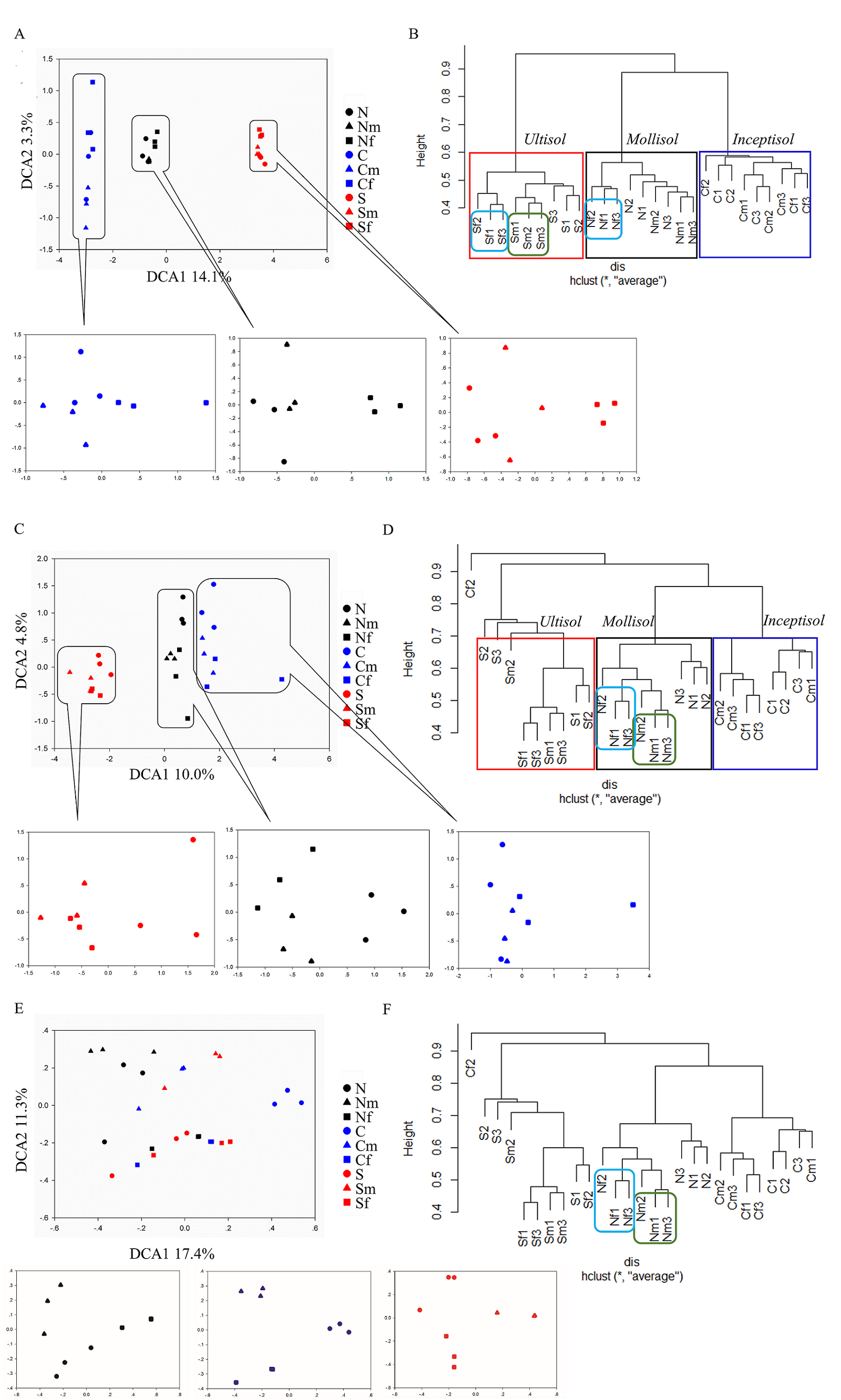

Supplement: Figure S1 [file sys004162038sf1.tif]

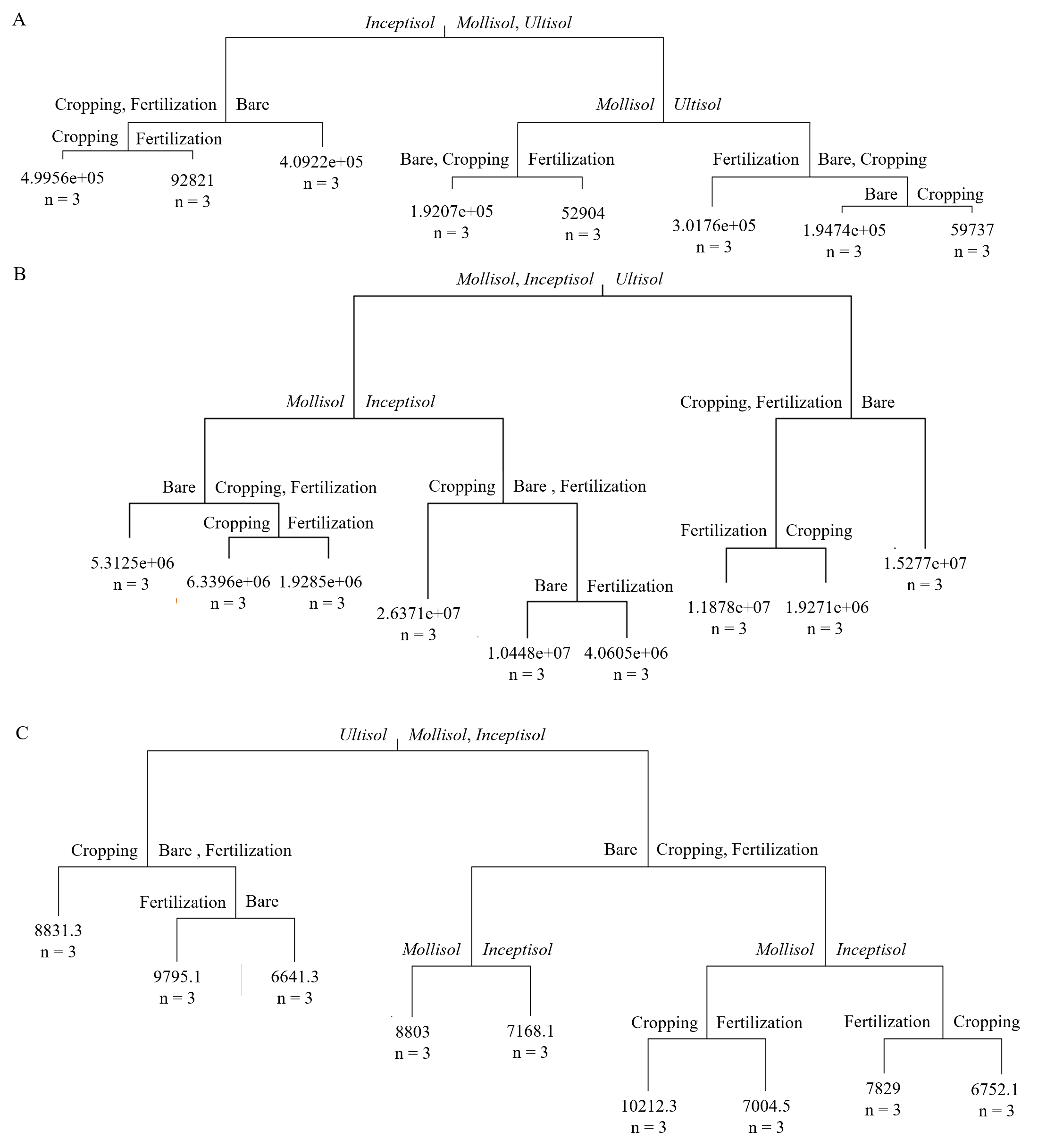

Supplement: Figure S2 [file sys004162038sf2.tif]

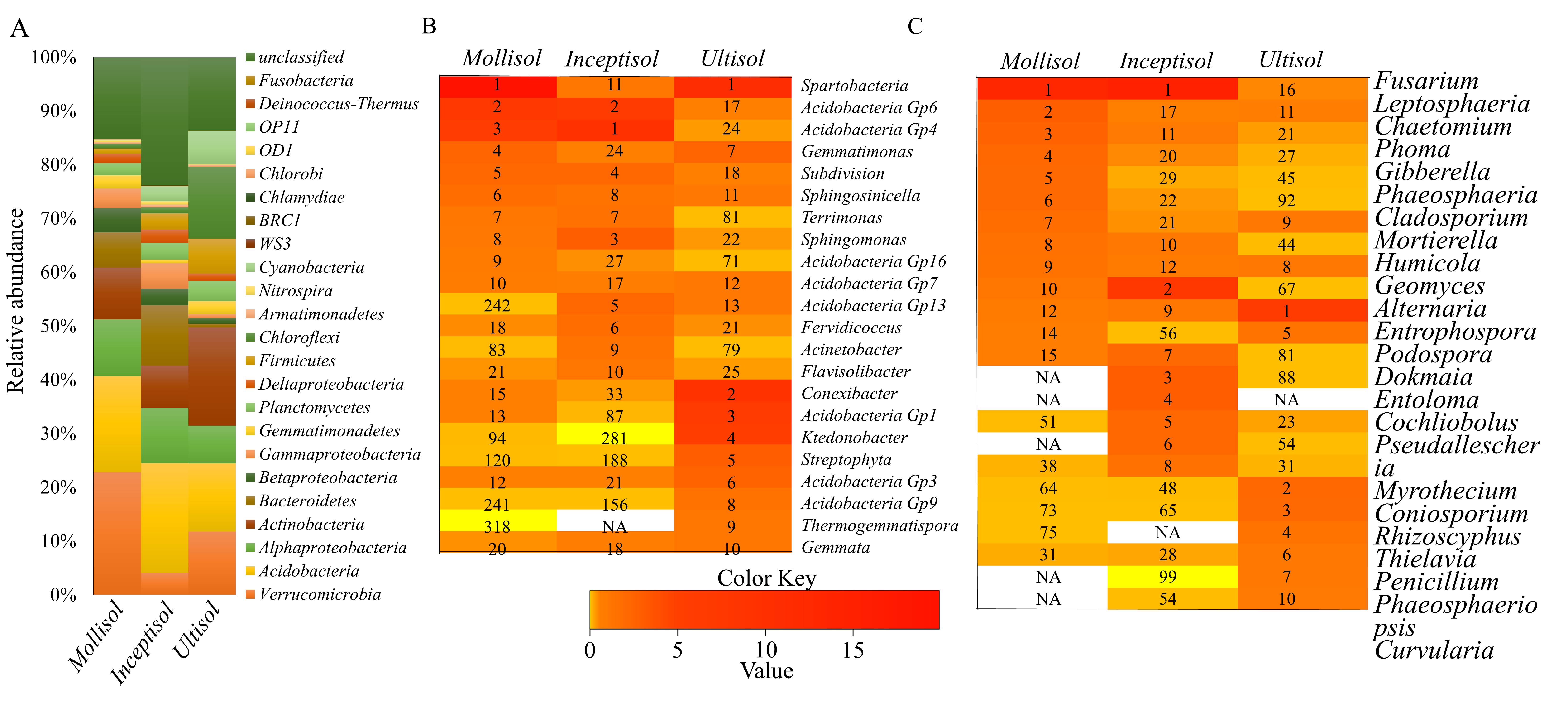

Supplement: Figure S3 [file sys004162038sf3.tif]

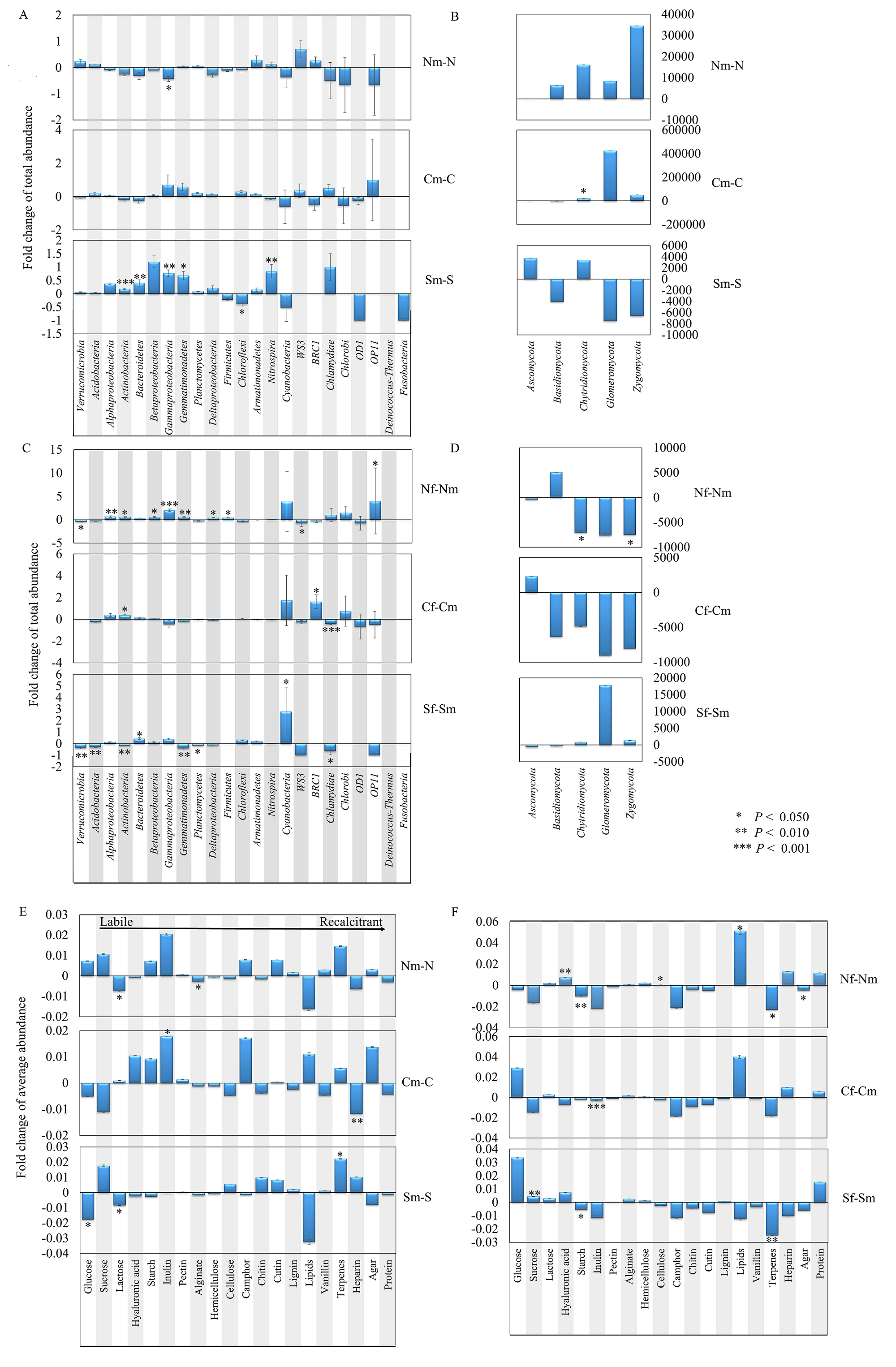

Supplement: Figure S4 [file sys004162038sf4.tif]

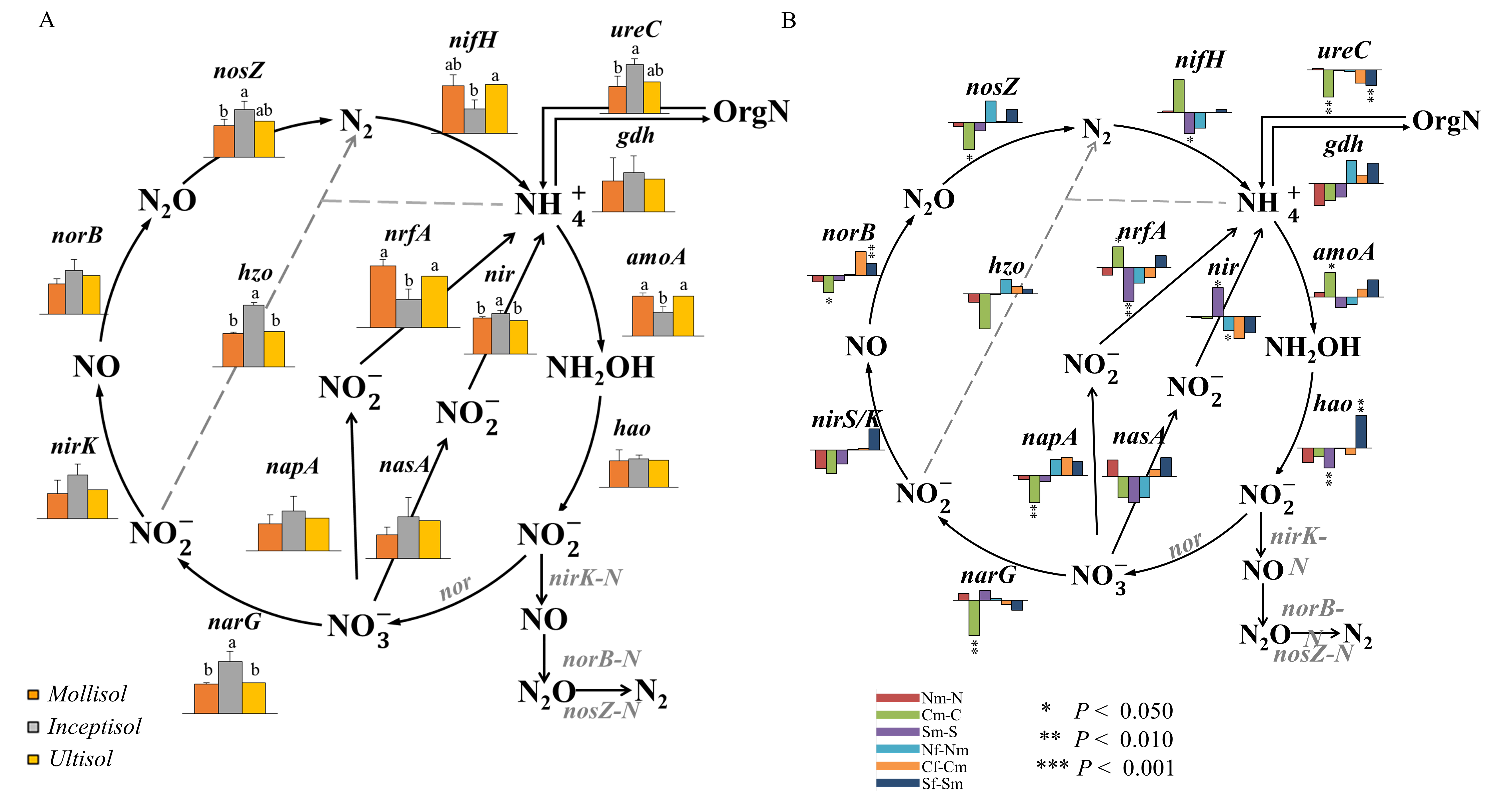

Supplement: Figure S5 [file sys004162038sf5.tif]

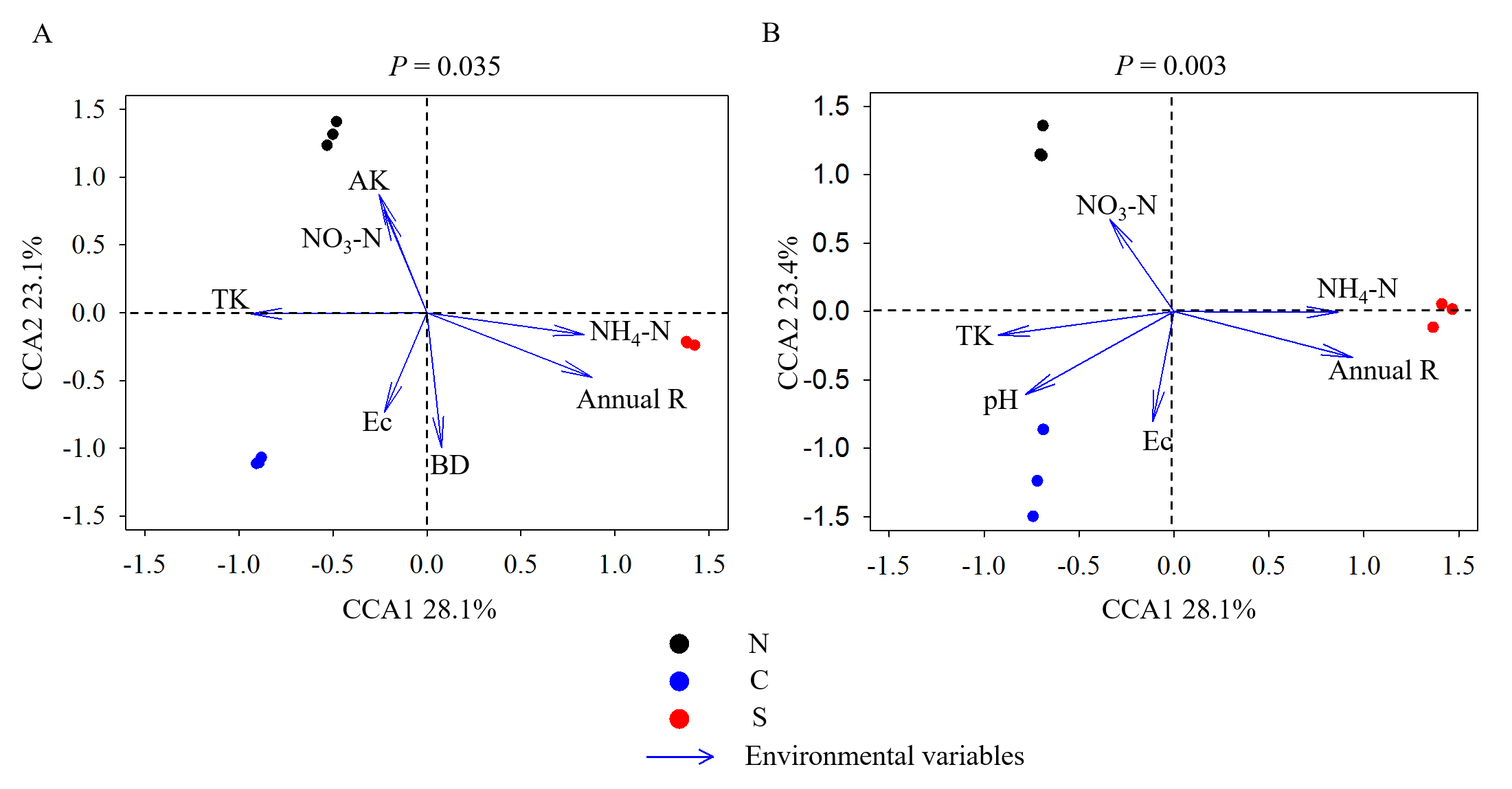

Supplement: Figure S6 [file sys004162038sf6.tif]

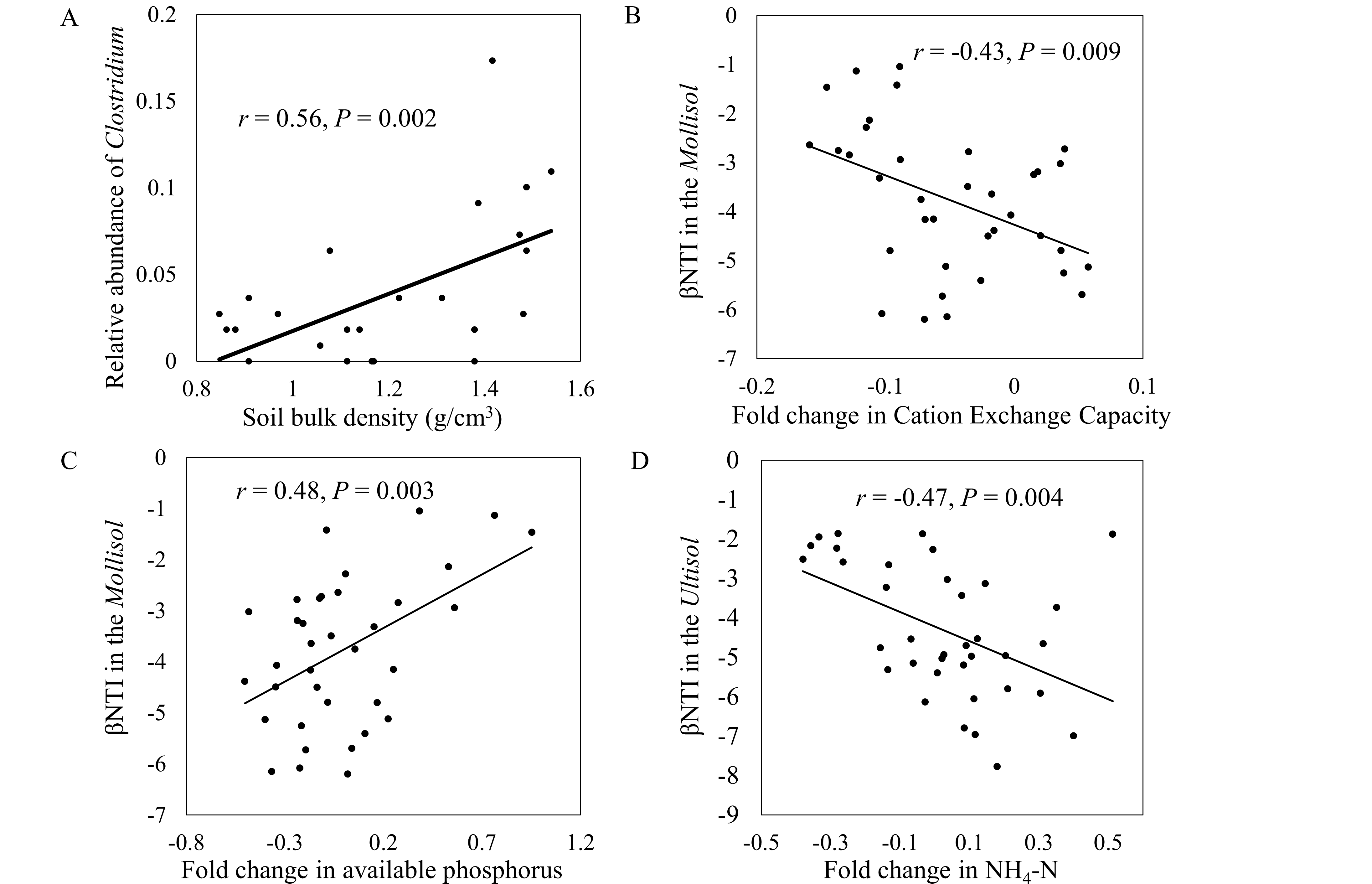

Supplement: Figure S7 [file sys004162038sf7.tif]
