# Supplementary material for: Zonal Soil Type Determines Soil Microbial Responses to Maize Cropping and Fertilization
Source: mSystems. 2016 Jul 12;1(4):e00075-16. doi: 10.1128/mSystems.00075-16 (PMC5069962; doi:10.1128/mSystems.00075-16)
Supplement: Table S2 [file sys004162038st9.docx]

**Table S2** Relative abundance of genus that consistently responded to maize cropping (*Gp7*) or fertilization (*Gp4*, *Gp6* and *Fusarium*)

| genus | N | Nm | Nf | C | Cm | Cf | S | Sm | Sf |
| --- | --- | --- | --- | --- | --- | --- | --- | --- | --- |
| *Gp4* | 5.66 ± 0.97b | 6.25 ± 0.75b | 2.94 ± 0.26c | 9.58 ± 2.41a | 10.34 ± 1.98a | 6.91 ± 1.42b | 0.31 ± 0.12d | 0.4 ± 0.11d | 0.1 ± 0.02d |
| *Gp6* | 7.69 ± 2.57ab | 8.92 ± 3.18a | 5.57 ± 1.44b | 6.08 ± 2.42ab | 7.89 ± 1.01ab | 6.7 ± 0.19ab | 0.61 ± 0.09c | 0.9 ± 0.24c | 0.5 ± 0.1c |
| *Gp7* | 0.75 ± 0.1c | 0.88 ± 0.25bc | 0.68 ± 0.17c | 0.71 ± 0.08c | 0.84 ± 0.08bc | 0.89 ± 0.07bc | 0.99 ± 0.17b | 1.29 ± 0.03a | 0.6 ± 0.15c |
| *Fusarium* | 12.92 ± 6.92a | 6.77 ± 1.8ab | 14.25 ± 6.71a | 14.92 ± 11.45a | 7.79 ± 3.57ab | 9.48 ± 6.62ab | 0.48 ± 0.47b | 0.97 ± 0.13b | 2.53 ± 1.58b |

Letters behind each value indicate significance of differences. Treatments with any same letters are insignificantly different (*P* > 0.05) as determined by one-way ANOVA followed by the LSD test in SAS version 6.1.
