# Supplementary material for: Zonal Soil Type Determines Soil Microbial Responses to Maize Cropping and Fertilization
Source: mSystems. 2016 Jul 12;1(4):e00075-16. doi: 10.1128/mSystems.00075-16 (PMC5069962; doi:10.1128/mSystems.00075-16)
Supplement: Table S3 [file sys004162038st10.docx]

**Table S3** Mantel tests to determine the significances of correlations between environmental variables and microbial communities in bare fallow soils

|  | Bacteria | | Fungi | |
| --- | --- | --- | --- | --- |
|  | *r* | *P* | *r* | *P* |
| pH | 0.84 | **0.001^b^** | 0.63 | **0.013** |
| SOM^a^ | 0.06 | 0.373 | 0.11 | 0.195 |
| WHC | 0.12 | 0.228 | 0.14 | 0.187 |
| BD | 0.25 | **0.042** | 0.19 | 0.108 |
| Sp | 0.20 | **0.052** | 0.26 | **0.050** |
| EC | 0.05 | 0.314 | -0.16 | 0.897 |
| CEC | 0.04 | 0.434 | 0.10 | 0.172 |
| TN | -0.03 | 0.475 | 0.06 | 0.244 |
| TP | 0.74 | **0.005** | 0.74 | **0.011** |
| TK | 0.78 | **0.005** | 0.77 | **0.006** |
| AP | 0.19 | 0.091 | 0.15 | 0.131 |
| AK | -0.08 | 0.675 | -0.02 | 0.483 |
| NH_4_-N | 0.51 | **0.013** | 0.49 | **0.015** |
| NO_3_-N | -0.13 | 0.888 | -0.04 | 0.533 |
| Annual T | 0.37 | **0.035** | 0.43 | **0.020** |
| Annual R | 0.77 | **0.009** | 0.83 | **0.001** |

*^a^*Abbreviation: SOM – soil organic matter, WHC – water hold capacity, BD – soil bulk density, Sp – soil porosity, EC – electrical conductivity, CEC – cation exchange capacity, TN – total nitrogen, TP – total phosphorus, TK – total potassium, AP – available phosphorus, AK - available potassium, Annual T – annual average temperature, Annual R – annual rainfall.

^a^Bold font indicates significant correlation of *P* < 0.050
